# Plasma Levels of Omega-3 and Omega-6 Derived Oxylipins Are Associated with Fecal Microbiota Composition in Young Adults

**DOI:** 10.3390/nu14234991

**Published:** 2022-11-24

**Authors:** Huiwen Xu, Lucas Jurado-Fasoli, Lourdes Ortiz-Alvarez, Francisco J. Osuna-Prieto, Isabelle Kohler, Xinyu Di, Ramiro Vilchez-Vargas, Alexander Link, Julio Plaza-Díaz, Angel Gil, Patrick C. N. Rensen, Jonatan R. Ruiz, Borja Martinez-Tellez

**Affiliations:** 1 PROFITH (PROmoting FITness and Health through Physical Activity) Research Group, Sport and Health University Research Institute (iMUDS), Department of Physical and Sports Education, Faculty of Sport Sciences, University of Granada, 18071 Granada, Spain; 2 Department of Biochemistry and Molecular Biology II, School of Pharmacy, University of Granada, 18071 Granada, Spain; 3 Department of Physiology, Faculty of Medicine, University of Granada, 18071 Granada, Spain; 4 Department of Analytical Chemistry, Faculty of Sciences, University of Granada, 18071 Granada, Spain; 5 Division of BioAnalytical Chemistry, Amsterdam Institute of Molecular and Life Sciences (AIMMS), Vrije Universiteit Amsterdam, 1081 Amsterdam, The Netherlands; 6 Center for Analytical Sciences Amsterdam, 1081 Amsterdam, The Netherlands; 7 Department of Systems Biomedicine and Pharmacology, Leiden Academic Centre for Drug Research (LACDR), Leiden University, 2300 Leiden, The Netherlands; 8 Department of Gastroenterology, Hepatology and Infectious Diseases, Otto-von-Guericke-University Magdeburg, 39106 Magdeburg, Germany; 9 Children’s Hospital of Eastern Ontario Research Institute, Ottawa, ON K1H 8L1, Canada; 10 Instituto de Investigación Biosanitaria, ibs.Granada, 18014 Granada, Spain; 11 CIBEROBN, Biomedical Research Networking Center for Physiopathology of Obesity and Nutrition, Carlos III Health Institute, 28029 Madrid, Spain; 12 Institute of Nutrition and Food Technology, Center of Biomedical Research, University of Granada, 18071 Granada, Spain; 13 Department of Medicine, Division of Endocrinology, and Einthoven Laboratory for Experimental Vascular Medicine, Leiden University Medical Center, 2300 Leiden, The Netherlands; 14 Department of Education, Faculty of Education Sciences and SPORT Research Group (CTS-1024), CERNEP Research Center, University of Almería, 04120 Almería, Spain

**Keywords:** gut microbiota, inflammation, intestinal alkaline phosphatase, microbiome, PUFAs

## Abstract

Pre-clinical studies suggest that circulating oxylipins, i.e., the oxidation products of polyunsaturated fatty acids (PUFAs), modulate gut microbiota composition in mice, but there is no information available in humans. Therefore, this study aimed to investigate the relationship between omega-3 and omega-6 derived oxylipins plasma levels and fecal microbiota composition in a cohort of young adults. 80 young adults (74% women; 21.9 ± 2.2 years old) were included in this cross-sectional study. Plasma levels of oxylipins were measured using liquid chromatography-tandem mass spectrometry. Fecal microbiota composition was analyzed by V3-V4 16S rRNA gene sequencing. We observed that plasma levels of omega-3 derived oxylipins were positively associated with the relative abundance of *Clostridium cluster IV* genus (*Firmicutes* phylum; rho ≥ 0.415, *p* ≤ 0.009) and negatively associated with the relative abundance of *Sutterella* genus (Proteobacteria phylum; rho ≥ −0.270, *p* ≤ 0.041), respectively. Moreover, plasma levels of omega-6 derived oxylipins were negatively associated with the relative abundance of *Acidaminococcus* and *Phascolarctobacterium* genera (*Firmicutes* phylum; all rho ≥ −0.263, *p* ≤ 0.024), as well as *Sutterella*, *Succinivibrio*, and *Gemmiger* genera (*Proteobacteria* phylum; all rho ≥ −0.263, *p* ≤ 0.024). Lastly, the ratio between omega-6 and omega-3 oxylipins plasma levels was negatively associated with the relative abundance of *Clostridium* cluster IV genus (*Firmicutes* phylum; rho = −0.334, *p* = 0.004) and *Butyricimonas* genus (*Bacteroidetes* phylum; rho = −0.292, *p* = 0.014). In conclusion, our results show that the plasma levels of omega-3 and omega-6 derived oxylipins are associated with the relative abundance of specific fecal bacteria genera.

## 1. Introduction

Chronic diseases, such as type 2 diabetes and cardiovascular diseases, usually accompany low-grade chronic inflammation [1,2,3]. The rising prevalence of such diseases is believed to be driven by an unbalanced diet, which is often characterized by a low intake of omega-3 polyunsaturated fatty acids (PUFAs) and a high intake of omega-6 PUFAs [4,5].

Omega-3 and omega-6 PUFAs can be oxidized into oxylipins by lipoxygenases (LOXs), cyclooxygenases (COXs), and the cytochrome P450 (CYP450) family enzymes [6,7]. Oxylipins are the primary mediators of the PUFA-related effects through their binding to G protein-coupled receptors (GPCRs) or peroxisome proliferator-activate receptors (PPARs) [6,7]. Generally, omega-3 derived oxylipins exert anti-inflammatory and pro-resolutive actions since both eicosapentaenoic acid (EPA) and docosahexaenoic acid (DHA) decrease the synthesis of pro-inflammatory mediators and increase the production of anti-inflammatory mediators (e.g., prostaglandins of the three series, leukotrienes of the five series, resolvins, protectins and maresins) [8,9]. In contrast, omega-6 derived oxylipins typically increase inflammation by acting as pro-inflammatory mediators (e.g., prostaglandins of the two series and leukotrienes of the four series) [9,10].

The gut microbiota is composed of microorganisms that colonize the gastrointestinal tract, where bacteria are the most abundant [11]. In humans, the gut microbiota is mainly composed of five phyla: Bacteroidetes, Firmicutes, Proteobacteria, Actinobacteria and Verrucomicrobia [12,13]. The gut microbiota is involved in the maintenance of the gut barrier permeability [14,15] and regulation of the intestinal inflammatory status through specific bacteria, their interaction with intestinal cell receptors and their secreted metabolites (e.g., short chain fatty acids) [16,17]. Interestingly, the composition of gut microbiota and metabolite production can be modified by dietary intake (e.g., nitrogenous compounds, carbohydrates, fatty acids, fiber) [18]. The specific dietary intake of PUFAs, concretely omega-3 PUFAs, increases *Bacteroidetes:Firmicutes* ratio and the diversity and counts of commensal bacteria (i.e., *Akkermansia muciniphila*, *Lactobacillus* and *Bifidobacterium*) [19]. In this sense, dietary patterns with a high content of omega-3 and a low content of omega-6 PUFAs (e.g., Mediterranean Diet) could have a positive influence on the health status of humans via the modulation of the gut microbiota partially due to the positive effects of these PUFAs in the intestinal immunity [19,20]. Therefore, studying the relationship between the primary mediators of PUFAs effects (e.g., oxylipins) with gut microbiota composition is of clinical interest.

A recent study showed that FAT-1 transgenic mice, which can convert omega-6 to omega-3 PUFAs, had a higher relative abundance of Firmicutes, Bacteroides, and Actinobacteria phyla in comparison to wild-type (WT) mice [21]. On the other hand, FAT-2 transgenic mice, which can transform monounsaturated fatty acids into omega-6 PUFAs, showed a distinct gut microbiota composition compared to FAT-1 mice [21]. Specifically, FAT-2 mice were characterized by a depletion of the *Bifidobacteriaceae* family (*Actinobacteria* phylum) vs. WT mice [22]. This study demonstrated that omega-6 and omega-3 PUFAs levels influence gut microbiota composition and their secreted metabolites, influencing intestinal permeability, reducing the inflammatory status and ultimately affecting the risk of chronic disease [22]. In addition, gut microbiota dysbiosis induced by a typical cafeteria diet altered the plasma profile of oxylipins in obese rats [23]. We, therefore, hypothesize that plasma levels of omega-3 and omega-6 derived oxylipins are associated with gut microbiota composition. In the present study, we investigated the association between omega-3 and omega-6 derived oxylipins plasma levels and fecal microbiota composition in young adults.

## 2. Materials and Methods

### 2.1. Participants

The participants were part of the ACTIBATE study [24], an exercise-based randomized controlled trial (ClinicalTrials.gov ID: NCT02365129) designed to evaluate the effect of exercise training on brown adipose tissue activity. Participants were recruited via advertisements in electronic media and leaflets. All participants reported being sedentary (less than 20 min of moderate/vigorous physical activity on less than three days per week) and had a stable body weight over the preceding three months (<3 kg change). The exclusion criteria were being pregnant, smoking, taking any medication, including antibiotics, and having an acute or chronic disease. The study protocol was designed following the Declaration of Helsinki. All participants gave their informed consent and were approved by the Ethics Committee on Human Research of the University of Granada (n°.924) and Servicio Andaluz de Salud (Centro de Granada, CEI-Granada).

In the present study, only participants with baseline data for plasma oxylipins and fecal microbiota composition available were included (80 young adults; *n* = 59 women; 21.9 ± 2.2 years old).

### 2.2. Determination of Plasma Levels of Oxylipins

Blood samples were collected between 8:00–9:00 AM after 10 h overnight fasting. All blood samples were immediately centrifuged to obtain plasma aliquots (obtained with Vacutainer^®^ Hemogard™ tubes, containing the potassium salt of ethylenediaminetetraacetic acid as an anticoagulant) and stored at −80 °C. Plasma levels of omega-3 and omega-6 oxylipins were measured with a targeted metabolomics approach using liquid chromatography-tandem mass spectrometry (LC-MS/MS), with a method validated according to the Food and Drug Administration (FDA) bioanalytical method validation guidelines [25].

With this targeted LC-MS/MS approach, the relative quantification of oxylipins derived from the conversion of the omega-3 PUFAs α-linolenic acid (ALA), eicosapentaenoic acid (EPA), and docosahexaenoic acid (DHA); as well as omega-6 PUFAs linoleic acid (LA), dihomo-γ-linolenic acid (DGLA), arachidonic acid (AA), and adrenic acid (AdrA) was performed. The oxylipins detected by this method and the internal standards used are listed in Table S1. The ratio between the peak area of each oxylipin and the peak area of its respective internal standard was used for the relative quantitation. All the data from omega-3 derived oxylipin species were summed; the same was performed for omega-6 derived oxylipins (Table S2). Additionally, the plasma omega-6/omega-3 oxylipins ratio was calculated by dividing the sum of plasma omega-6 derived oxylipins peak area ratio by the sum of plasma omega-3 derived oxylipins peak area ratio. The methodology is described in detail in the supplementary material.

### 2.3. Fecal Collection and DNA Extraction

Fecal samples were stored in a 60-mL sterile plastic container, transported in a portable cooler to the research center and stored at −80 °C until DNA extraction. DNA extraction and purification were performed with a QIAamp DNA Stool Mini Kit (QIAGEN, Barcelona, Spain), according to the manufacturer’s instructions. Concentration and quality were determined with a NanoDrop ND1000 spectrophotometer (Thermo Fisher Scientific, DE, Waltham, Massachusetts, USA).

DNA was amplified by PCR targeting the V3 and V4 hypervariable regions of the bacterial 16S rRNA gene. The amplicons were sequenced in a MiSeq (Illumina, San Diego, CA, USA), using the Illumina MiSeq paired-end sequencing system (2 × 300 nt) (Illumina, San Diego, CA, USA). We used the “dada2” package version 1.10.1 in R software [26] for merging and filtering raw sequences (FastQ files). Ribosomal Data Project (RDP) [27] was used to assign the phylotypes to their specific taxonomic affiliation (from phylum to genus). The methodology is described in detail in the supplementary material.

### 2.4. Anthropometry and Body Composition

The SECA model 799 electronic column scale and stadiometer (SECA, Hamburg, Germany) were used to measure the height and weight of study participants wearing standard clothes without shoes. A dual-energy X-ray absorptiometry scan (Hologic Discovery Wi Marlborough, MA) was used to measure body composition, i.e., lean and fat mass. The body mass index (BMI) was calculated as weight/height^2^.

### 2.5. Dietary Assessment

The dietary assessment is explained in detail elsewhere [28,29,30]. The EvalFINUT^®^ software was used to assess dietary intake (energy and nutrient intake) from three 24 h dietary recalls [28,29,30]. The 24 h dietary recalls were undertaken on three separate days (i.e., two weekdays and one weekend) during face-to-face interviews by qualified and trained dietitians. Two dieticians independently introduced the data from all interviews in the software. The food frequency consumption was assessed using a food frequency questionnaire (FFQ) [31], in which participants answered how often they had consumed each food item over the last three months, using commonly used portion size.

### 2.6. Statistical Analysis

Sample size estimation and power calculation were based on the primary outcome of the ACTIBATE study [24]. The current study is based on a secondary analysis using its baseline data. Therefore, no specific power calculation or sample size estimation was developed for the present study.

The descriptive parameters are presented as mean and standard deviation. First, data normality was checked using the D’Agostino & Pearson test. Due to the non-normal distribution of plasma levels of oxylipins and parameters of fecal microbiota composition, all analyses were conducted using non-parametric tests.

To investigate the relationship between the sum of omega-3 and omega-6 derived oxylipins and the omega-6/omega-3 oxylipin ratio with fecal microbiota composition at phylum and genus taxonomy levels, we employed Spearman correlation analysis by using “psych” and “corrplot” packages in R software. Volcano plots were used to depict these correlations using GraphPad Prism software (GraphPad Software, San Diego, CA, USA, version 8.0.0). Then, to examine the relationship between plasma levels of each omega-3 and omega-6 oxylipin and specific bacterial microbiota, we used Spearman correlation analysis, using “psych” and “corrplot” in R software. Heatmap plots were used to represent these correlations using the “Gplot” package in R software. Lastly, partial Spearman correlations were adjusted for BMI, total PUFAs, and fish intake. The level of significance was set at *p* < 0.05.

## 3. Results

### 3.1. Characteristics of the Study Participants

Table 1 shows the descriptive characteristics of the study participants (74% women; 21.9 ± 2.2 years old).

### 3.2. The Plasma Levels of Omega-3 Oxylipins Are Associated with the Relative Abundance of Clostridium Cluster IV and Sutterella Genera

**Figure 1:**
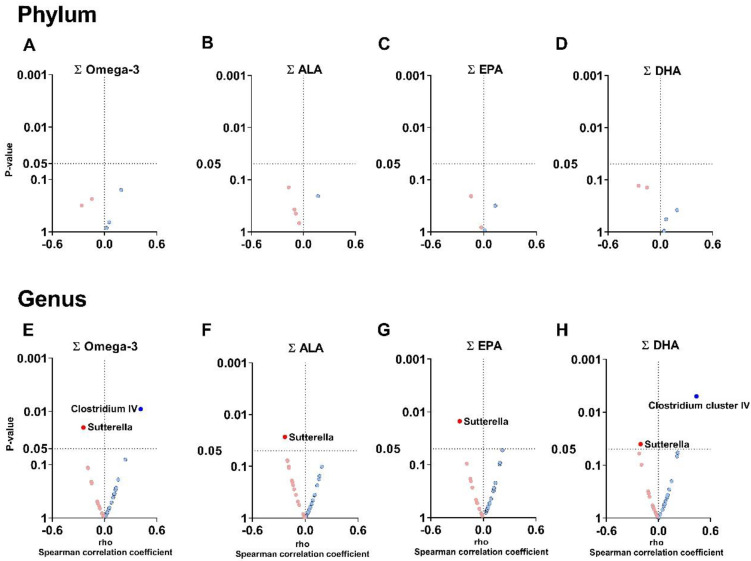
Volcano plots show associations between plasma levels of omega-3 oxylipins and the relative abundance of fecal microbiota composition at the phylum and genus levels. Total omega-3 (**A**,**E**), ALA- (**B**,**F**), EPA- (**C**,**G**), and DHA- (**D**,**H**) derived oxylipins were computed from the individual peak area ratio of each oxylipin within each group. The X-axis represents Spearman’s correlation coefficients, whereas the Y-axis represents the *p* values of the correlations. Only significant associations (*p* < 0.05) were annotated with the name of the phylum or genus. Blue dots represent positive associations, whereas red dots represent negative associations. Abbreviations: ALA: α-linolenic acid; DHA: docosahexaenoic acid; EPA: eicosapentaenoic acid.

At the phylum level, we found no association between the sum of plasma levels of omega-3 oxylipins with fecal microbiota composition (all *p* > 0.05; Figure 1A–D). However, at the genus level, the total plasma levels of both omega-3 and DHA-derived oxylipins (Table S3) were positively associated with the relative abundance of *Clostridium cluster IV* (*Firmicutes* phylum; rho ≥ 0.415, *p* ≤ 0.009; Figure 1E,H). Moreover, the total plasma levels of omega-3, ALA-, EPA-, and DHA-derived oxylipins were negatively associated with the relative abundance of the *Sutterella* genus (*Proteobacteria* phylum; rho ≥ −0.270, *p* ≤ 0.041; Figure 1E–H).

The plasma levels of the individual omega-3 oxylipins DPA, DHA, 8-HDoHE, 13-HDoHE and 19,20-DiDHPA, were positively associated with the relative abundance of *Clostridium cluster IV* genus (all rho ≥ 0.314, *p* ≤ 0.018; Figure S1A). Conversely, the plasma levels of ALA, EPA, 5-HEPE, DHA, 4-HDoHE and 19,20-DiDHPA were negatively associated with the relative abundance of the *Sutterella* genus (all rho ≥ −0.338, *p* ≤ 0.042; Figure S1B). These associations remained significant after adjusting for BMI, PUFAs, and fish intake (Tables S3 and S4).

### 3.3. The Plasma Levels of Omega-6 Oxylipins Are Negatively Associated with the Relative Abundance of Genera Belonging to Firmicutes and Proteobacteria Phyla

**Figure 2:**
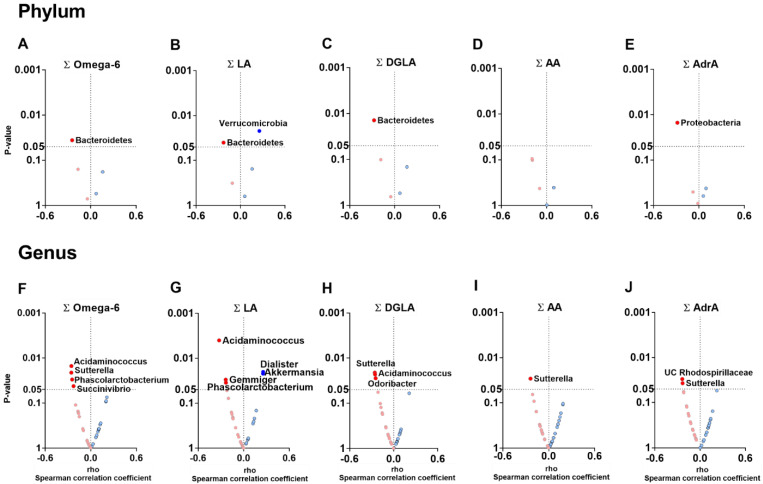
Volcano plots showing associations between the plasma levels of omega-6 oxylipins and the relative abundance of fecal microbiota composition at phylum and genus levels. Total omega-6 (**A**,**F**), LA- (**B**,**G**), DGLA- (**C**,**H**), AA- (**D**,**I**), and AdrA- (**E**,**J**) derived oxylipins groups were computed from the individual area peak ratio of each oxylipins group. The X-axis represents Spearman’s correlation coefficients, whereas the Y-axis represents the *p* values of the correlations. Only significant correlations (*p* < 0.05) were annotated with the name of the phylum or genus. Blue dots represent positive correlations, whereas red dots represent negative correlations. Abbreviations: AA: arachidonic acid; AdrA: adrenic acid; DGLA: dihomo-γ-linolenic acid; LA: linoleic acid; UC: unclassified.

At the phylum level, the total plasma levels of omega-6, LA- and DGLA-derived oxylipins were negatively associated with the relative abundances of *Bacteroidetes* (all rho ≥ −0.274, *p* ≤ 0.040; Figure 2A–C), whereas the total plasma levels of AdrA-derived oxylipins were negatively associated with the relative abundance of *Proteobacteria* (rho = −0.284, *p* = 0.013; Figure 2E). However, the total plasma levels of LA-derived oxylipins were positively associated with the relative abundance of *Verrucomicrobia* phylum (rho = 0.255, *p* = 0.022; Figure 2B). At the genus level, we found that the total plasma levels of omega-6, LA-, and DGLA-derived oxylipins were negatively associated with the relative abundance of Acidaminococcus and *Phascolarctobacterium* genera (*Firmicutes* phylum; all rho ≥ −0.326, *p* ≤ 0.034; Figure 2F–H). We also observed that the total plasma levels of omega-6, DGLA-, AA-, and AdrA-derived oxylipins levels were negatively associated with the relative abundance of *Sutterella*, *Succinivibrio*, and *Gemmiger* genera (*Proteobacteria* phylum; all rho ≥ −0.258, *p* ≤ 0.042; Figure 2F–J). Similarly, the total plasma levels of DGLA-derived oxylipins were negatively associated with the relative abundance of the *Odoribacter* genus (*Bacteroidetes* phylum; rho = −0.246, *p* = 0.028; Figure 2H).

The plasma levels of individuals omega-6 oxylipins LA, DGLA, AA, 5-HETE and AdrA, were negatively associated with the relative abundance of the *Sutterella* genus (all rho ≥ −0.313, *p* ≤ 0.027; Figure S1B). All these associations remained significant after adjusting for BMI, PUFA, and fish intake (Tables S3 and S4).

### 3.4. The Plasma Omega-6/Omega-3 Oxylipin Ratio Is Negatively Associated with the Relative Abundance of Clostridium Cluster IV and Butyricimonas Genera

**Figure 3:**
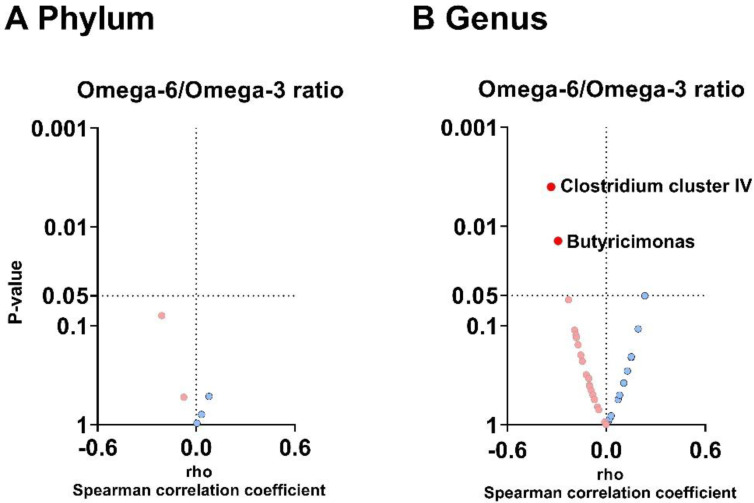
Volcano plots showing the associations between the omega-6/omega-3 oxylipin ratio in plasma and the relative abundance of fecal microbiota composition at the phylum (**A**) and genus levels (**B**). The X-axis represents Spearman’s correlation coefficients, whereas the Y-axis represents the *p* values of the correlations. Only significant correlations (*p* < 0.05) were annotated with the name of the phylum or genus. Blue dots represent positive correlations, whereas red dots represent negative correlations.

At the phylum level, we found no associations between the omega-6/omega-3 oxylipin ratio in plasma and fecal microbiota composition (all *p* > 0.05; Figure 3A). On the other hand, at the genus level, the omega-6/omega-3 oxylipin ratio was negatively associated with the relative abundance of *Clostridium cluster IV* (*Firmicutes* phylum; rho = −0.334, *p* = 0.004; Figure 3B) and *Butyricimonas* genera (*Bacteroidetes* phylum; rho = −0.292, *p* = 0.014; Figure 3B) independently of BMI, PUFA, and fish intake (Table S3).

## 4. Discussion

In the present study, we show that plasma levels of oxylipins are linked to the fecal microbiota composition of young adults. Specifically, plasma levels of omega-3 derived oxylipins were positively associated with the relative abundance of *Clostridium* cluster IV and negatively associated with the relative abundance of *Sutterella* genera. Additionally, plasma levels of omega-6 derived oxylipins were negatively associated with the relative abundance of several genera. In contrast, the ratio of omega-6/omega-3 oxylipins in plasma was negatively associated with the relative abundance of *Clostridium* cluster IV and *Butyricimonas* genera. These findings suggest that the plasma levels of omega-3 and omega-6 derived oxylipins may modulate the relative abundance of gut microbiota composition, or vice versa, in young adults.

We found that the plasma levels of omega-3 derived oxylipins and the omega-6/omega-3 oxylipin ratio in plasma are positively and negatively associated, respectively, with the relative abundance of *Clostridium* cluster IV genus, which is composed of *Clostridium*, *Eubacterium*, *Ruminococcus*, and *Anaerofilum* genera. Similarly, the *FAT-1* mice, capable of transforming omega-6 into omega-3 PUFAs, also showed a higher relative abundance of *Clostridium* cluster IV genus compared to WT mice [21]. The *Clostridium* cluster IV genus bacteria have been shown to exert anti-inflammatory effects and maintain intestinal health via the production of short-chain fatty acids, such as butyrate [32]. These findings suggest that higher plasma levels of omega-3 derived oxylipins could improve systemic and intestinal health through an increased relative abundance of *Clostridium* cluster IV genus, which produces butyrate and ultimately enhances intestinal barrier function and immunity [33]. In contrast, gut microbiota influences the production of omega-6 oxylipins, conferring host resistance to high-fat diet-induced obesity and adipose tissue inflammation in mice [34]. The above-mentioned pre-clinical studies are confirmed in the present human cohort, reinforcing the clinical benefits of omega-3 PUFAs, specifically its oxylipins, on human health.

Additionally, we identified that plasma levels of omega-3 and omega-6 oxylipins are negatively associated with the relative abundance of the *Sutterella* genus in feces. In line with these results, rats consuming a diet rich in the omega-6 PUFA LA for 10 weeks showed a decrease in the relative abundance of the *Sutterella* genus [35]. In contrast, daily oral supplementation with omega-3 PUFAs (i.e., 4 g of EPA and DHA) for eight weeks increased the relative abundance of *Sutterellaceae* family in feces of in middle-aged healthy humans [36]. After a wash-out period of eight weeks, the relative abundance of the *Sutterellaceae* family returned to baseline levels [36]. These studies show that supplementing omega-3 or omega-6 PUFAs can modulate the relative abundance of the specific bacteria in the gut. This effect might be explained by the direct effects of dietary PUFAs in the intestine since PUFAs and their downstream oxylipins can modify the fatty acid composition of the intestinal brush border membrane, increasing the intestinal alkaline phosphatase (IAP) activity in the small intestine [37,38]. Thus, we hypothesize that oxylipins could modulate IAP activity similarly to their PUFA precursors. In this regard, both supplementation of omega-3 PUFAs [21] and overproduction of omega-6 PUFAs [39] have been shown to induce the release of IAP from enterocytes, either through the modulation of resolvins (i.e., distal mediators of the anti-inflammatory/pro-resolution cascade derived from omega-3 oxylipins) [40] or the increase in the relative abundance of certain bacteria (e.g., *Sutterella* genus [22] that can produce lipopolysaccharides [LPS]). IAP dephosphorylates LPS [41], preventing LPS from binding to Toll-like receptor-4 (TLR4) [41], consequently avoiding the activation of the inflammatory cascade [41]. Since gut microbiota could contribute to the levels of oxylipins in response to inflammation [23], we hypothesize that plasma oxylipins could translocate to the gut lumen, as with other inflammatory mediators. Therefore, the relationship observed between omega-3 and omega-6 oxylipins plasma levels and the relative abundance of the *Sutterella* genus could be partially explained by the modulation of IAP activity in humans. Although this hypothesis is based on pre-clinical studies, it should be explored in future studies specifically designed. However, our results describe the relationship between the omega-3 and omega-6 oxylipins with these specific bacteria for the first time in humans. Since oxylipins are the primary mediators of PUFAs physiological effects, mainly in regulating the inflammatory and immune response, our results are of great clinical interest.

### Limitations and Strengths

This study shows the following limitations: the cross-sectional study design does not allow to establishment a cause-effect relationship. Our results should be interpreted with caution due to the lack of specific sample size and power calculation for the current study. The analyses were not controlled for false discovery rate (FDR) since this approach is hypothesis-generating, and adjusting for FDR might overcorrect potentially meaningful findings. Lastly, while these results generate new hypotheses, further research is needed to elucidate the direction of the crosstalk between plasma levels of oxylipins and fecal microbiota since plasma oxylipins might influence fecal microbiota composition and vice versa. On the other hand, two major strengths in this study are: (i) we performed DNA sequencing with one of the latest technologies (*Illumina* platform) and using the DADA2 program that uses amplicon sequence variants instead of operational taxonomic units [42]; and (ii) RDP conducted the annotation step until the genus taxon, a methodology with an annotation error less than 10% [43].

## 5. Conclusions

Our study reveals that plasma omega-3 oxylipins are positively associated with the relative abundance of *Clostridium cluster* IV genus. In contrast, plasma levels of omega-3 and omega-6 oxylipins are negatively associated with the relative abundance of the *Sutterella* genus in fecal samples of young adults. These results may suggest that plasma levels of omega-3 and omega-6 oxylipins modulate human gut microbiota composition. Based on the well-known influence of omega-3 and omega-6 oxylipins on human health, our results suggest that these oxylipins may be involved in regulating the gut barrier function in humans. However, future studies should address the influence of omega-3 and omega-6 supplementation in gut microbiota health, as well as the effect of different probiotics on the regulation of oxylipins in the plasma and the gut barrier.

## Figures and Tables

**Table 1:** Characteristics of the study participants.

	N	Mean	±	SD
Sex (women, %)	80	59 (73.8%)		
Age (years)	80	21.9	±	2.2
Body composition				
Lean mass (kg)	80	41.0	±	8.9
Fat mass (kg)	80	36.3	±	7.8
Body mass index (kg/m^2^)	80	24.7	±	4.7
Dietary intake				
Energy (kcal/day)	80	1920	±	489
PUFAs (g/day)	80	13	±	5
Fish (servings/week)	77	5	±	3
Total plasma levels of derived-oxylipins (peak area ratio)				
Omega-3	80	172.8	±	65.6
ALA	80	12.0	±	5.1
EPA	80	19.0	±	10.3
DHA	80	141.7	±	54.6
Omega-6	80	99.5	±	27.8
LA	80	28.6	±	10.6
DGLA	80	1.0	±	0.5
AA	80	66.4	±	22.2
AdrA	80	1.0	±	0.6
Omega-6/omega-3 oxylipin ratio	80	0.6	±	0.1
Fecal microbiota composition (phylum, %)				
*Actinobacteria*	80	1.6	±	1.6
*Bacteroidetes*	80	39.6	±	9.0
*Firmicutes*	80	48.8	±	9.7
*Proteobacteria*	80	6.5	±	4.8
*Verrucomicrobia*	80	2.3	±	4.3

Data are presented as mean and standard deviation (SD), otherwise stated. Total oxylipins in each group from the individual peak area ratio of each oxylipin; AA: arachidonic acid; AdrA: adrenic acid; ALA: α-linolenic acid; DHA: docosahexaenoic acid; DGLA: dihomo-γ-linolenic acid; EPA: eicosapentaenoic acid; LA: linoleic acid; PUFAs: polyunsaturated fatty acids.

## Data Availability

The data supporting this study’s findings are available from the corresponding author upon reasonable request, as the study consists of a high number of participants and outcomes and requires specific knowledge for data interpretation.

## Supplementary Materials

The following supporting information can be downloaded at: https://www.mdpi.com/article/10.3390/nu14234991/s1, Table S1: Overview of the labelled internal standards used in the LC-MS/MS method. Table S2: Overview of the detected omega-3 and omega-6 oxylipins, including the variability observed in QC samples (expressed using RSD). Analytes showing an RSD < 30% in the QC samples were kept for further analysis. Table S3: Partial Spearman correlation between total omega-3 and omega-6 oxylipins and the relative abundance of fecal microbiota composition at the genus level. Table S4: Partial Spearman correlation between plasma levels of individual omega-3 and omega-6 oxylipins and the relative abundance of fecal microbiota composition at the genus level. Figure S1: Spearman correlations between plasma levels of individual omega-3 (Panel A) and omega-6 (Panel B) oxylipins and the abundance relative of Clostridium IV and Sutterella genera [44,45,46].
