# Supplementary material for: Plasma Levels of Omega-3 and Omega-6 Derived Oxylipins Are Associated with Fecal Microbiota Composition in Young Adults
Source: Nutrients. 2022 Nov 24;14(23):4991. doi: 10.3390/nu14234991 (PMC9736377; doi:10.3390/nu14234991)
Supplement: Supplementary file 1 [file nutrients-14-04991-s001.zip › nutrients-2022265-supplementary.pdf]

Plasma levels of omega-3 and omega-6 derived oxylipins are associated with fecal microbiota composition in young adults

SUPPLEMENTARY METHODS

1. Determination of the plasma levels of oxylipins

1.1. Sample preparation

The sample preparation was performed under ice-cold conditions to prevent analyte degradation, except for the evaporation step[1]. Target analytes were extracted using liquid-liquid extraction. Before the extraction, 150 µL of plasma sample was transferred into 1.5 mL Eppendorf tubes and mixed with 5 µL of an antioxidant solution composed of 0.4 mg/mL of butylated hydroxytoluene (BHT) and 10 µL of an internal standard mix containing the isotopically-labeled analogs (**Table S2**). Then, 150 µL of buffer solution (0.2 M citric acid and 0.1 M disodium hydrogen phosphate at pH 4.5) were added, followed by the addition of 1000 µL of the extraction solvent composed of methyl tert-butyl ether (MTBE) and butanol (50:50, *v/v*). The samples were then mixed for 5 min using a bullet blender (Next Advance, Averill Park, NY) and centrifuged (16,000 g, 10 min, 4 °C). Next, 900 µL of supernatants were transferred to new 1.5 mL Eppendorf tubes and evaporated to dryness using a SpeedVac system at room temperature. The dry residues were reconstituted in 50 µL of a mixture of methanol:acetonitrile (70:30, *v/v*), and the reconstituted samples were centrifuged (16,000 g, 10 min, 4 °C). Finally, 40 µL of supernatant was transferred into autosampler vials with glass inserts for injection in the LC-MS/MS system.

1.2. *Liquid chromatography - tandem mass spectrometry analysis*

LC-MS/MS analysis was performed as previously described[2]. Briefly, the extracted samples were analyzed using a Shimadzu LC system (Shimadzu Corporation, Kyoto, Japan) coupled with a SCIEX QTRAP 6500+ mass spectrometer (SCIEX, Framingham, MA). Separation was carried out with a BEH C18 column (50 mm × 2.1 mm, 1.7 µm) from Waters Technologies (Milford, MA, USA) kept at 40°C. The mobile phase consisted

of 0.1% acetic acid in water (A), 0.1% acetic acid in acetonitrile/methanol (90:10, *v/v*, B) and 0.1% acetic acid in isopropanol (C). Electrospray ionization was performed in negative mode. For the MS/MS acquisition, a selected reaction mode (SRM) was used. SRM transitions were individually optimized for targeted analytes and respective internal standards using standard solutions.

1.3. Data pre-processing

For each target compound, SCIEX OS-MQ Software was used to calculate the ratio between its peak area and the peak area of its respective internal standard. The quality of the data was monitored using regular injection of quality control (QC) samples, consisting of blank plasma samples. QC samples were used to correct for between batch variations, using the in-house developed mzQuality workflow (available at http://www.mzQuality.nl)[3]. Relative standard deviations (RSDs) were calculated for the analytes present in the QC samples, analytes with RSD >30% were excluded (**Table S1**).

2. Fecal collection and DNA extraction

2.1. *Sequencing*

DNA was amplified by PCR, using the following primer pairs: 16S Amplicon PCR Forward Primer: 5´CCTACGGGNGGCWGCAG; and 16S Amplicon PCR Reverse Primer: 5′GACTACHVGGGTATCTAATCC targeting the V3 and V4 hypervariable regions. PCR assays were conducted in a final volume of 25 µL including: 12.5 µL of the 2X KAPA HiFi Hotstart prepared mixture (KAPA Biosystems, Woburn, MA, USA), 5 µL of each forward and reverse primer (1 µM), and 2.5 µL of DNA extracted (10 ng). PCR program: (i) denaturation at 95°C for 3 min, (ii) 8 denaturation cycles at 95°C for 30 s, (iii) annealing at 55°C for 30 s, (iv) elongation at 72°C for 30 s, (v) a final extension at 72°C for 5 min. Next, AMPure XP microspheres (Beckman Coulter, Indianapolis, IN, USA) were used to purify the 16S V3 and V4 amplicon away from free primers and

primer-dimer species. For the PCR index step, we used the Nextera XT index kit (Illumina, San Diego, CA, USA) to tag DNA with the sequencing adapters. The pooled PCR products were purified using AMPure XP balls (Beckman Coulter, Indianapolis, IN, USA) before quantification. The amplicons were sequenced in a MiSeq (Illumina, San Diego, CA, USA), using the Illumina MiSeq paired-end sequencing system (2x300nt) (Illumina, San Diego, CA, USA).

2.2.Bioinformatics

The "*dada2"* package version 1.10.1 in *R* software[4] was used for merging and filtering raw sequences (FastQ files). All samples that were above the 10,000 reads cut-off point were considered valid for the analyses. The samples were resampled to an equal sequencing depth using the "*phyloseq*"[5] package in *R* software (30,982 reads), obtaining a total of 11,158 different phylotypes.

Phylotypes were assigned to their specific taxonomic affiliation (from phylum to genus) based on the naive Bayesian classification with a pseudo-bootstrap threshold of 80% [5] using the "*Classifier"* function in Ribosomal Data Project (RDP)[6]. We obtained 16 phyla, which include 209 genera. For the main analysis, we performed the analyses if the average of the sequence abundance was higher than 1% and we used relative abundances (reads percent of each phylotype relative to the total number of reads).

REFERENCES

1. Di Zazzo A, Yang W, Coassin M, et al. Signaling lipids as diagnostic biomarkers for ocular surface cicatrizing conjunctivitis. *J Mol Med.* 2020;98(5):751-760. doi:10.1007/s00109-020-01907-w
2. Jurado-Fasoli L, Di X, Kohler I, et al. Omega-6 and omega-3 oxylipins as potential markers of cardiometabolic risk in young adults. *Obesity (Silver*

Spring). 2022;30(1):50-61. doi:10.1002/OBY.23282

3. Van Der Kloet FM, Bobeldijk I, Verheij ER, Jellema RH. Analytical error reduction using single point calibration for accurate and precise metabolomic phenotyping. *J Proteome Res*. 2009;8(11):5132-5141. doi:10.1021/pr900499r

4. R Core Team. R: A Language and Environment for Statistical Computing. Published online 2019.

5. McMurdie PJ, Holmes S. Phyloseq: An R Package for Reproducible Interactive Analysis and Graphics of Microbiome Census Data. *PLoS One*. 2013;8(4). doi:10.1371/journal.pone.0061217

6. Cole JR, Wang Q, Fish JA, et al. Ribosomal Database Project: Data and tools for high throughput rRNA analysis. *Nucleic Acids Res*. 2014;42(D1). doi:10.1093/nar/gkt1244

Table S1 Overview of the labeled internal standards used in the LC-MS/MS method.

**Abbreviation**	**Name (International Union of Pure and Applied Chemistry, IUPAC)**
Arachidonic acid-d_8 (C20:4-w6-d_8)	5*Z*,8*Z*,11*Z*,14*Z*-eicosatetraenoic acid-d_8
Docosahexaenoic acid-d_5 (C22:6-w3-d_5)	4*Z*,7*Z*,10*Z*,13*Z*,16*Z*,19*Z*-docosahexaenoic acid-d_5
Linoleic acid-d_4 (C18:2-w6-d_4)	9Z,12Z-octadecadienoic acid-d_4
d_{11}-5-iPF_2a-VI	(8β)-5,9α,11α-trihydroxy-prosta-6*E*,14*Z*-dien-1-oic acid-d_{11}
d_4-8-iso-PGE_2	9-oxo-11α,15*S*-dihydroxy-(8β)-prosta-5*Z*,13*E*-dien-1-oic acid-d_4
d_4-8-iso-PGF_2α	9α,11α,15*S*-trihydroxy-(8β)-prosta-5*Z*,13*E*-dien-1-oic acid-d_4
d_4-PGD_2	9α,15*S*-dihydroxy-11-oxo-prosta-5*Z*,13*E*-dien-1-oic acid-d_4
d_4-PGF_2α	9*S*,11*R*,15*S*-trihydroxy-5*Z*,13*E*-prostadienoic acid-d_4
d_9-PGE_2	9-oxo-11*R*,15*S*-dihydroxy-5*Z*,13*E*-prostadienoic acid-d_9
d_4-iPF_2α-IV	(8*S*)-10-[(1*R*,2*S*,3*S*,5*R*)-3,5-Dihydroxy-2-pentylcyclopentyl]-8-hydroxydeca-5,9-dienoic acid-d_4
d_{11}-8,12-iPF_2α -VI	(12α)-5,9α,11α-trihydroxy-prosta-6*E*,14*Z*-dien-1-oic acid-d_{11}
d_{17}-10-Nitrooleate	10-nitro,9Z,12*Z*-octadecadienoic acid-d_{17}
d_{11}-14,15-DiHETrE	14,15-dihydroxy-5*Z*,8*Z*,11*Z*-eicosatrienoic acid-d_{11}
d_4-9(S)-HODE	9*S*-hydroxy-10*E*,12*Z*-octadecadienoic acid-d_4
d_4-LTB_4	5*S*,12*R*-dihydroxy-6Z,8*E*,10*E*,14*Z*-eicosatetraene-1,20-dioic acid-d_4
d_4-TXB_2	9*S*,11,15*S*-trihydroxy-thromboxa-5*Z*,13*E*-dien-1-oic acid-d_4
d_6-20-HETE	20-hydroxy-5*Z*,8*Z*,11*Z*,14*Z*-eicosatetraenoic acid-d_6
d_8-12(S)-HETE	12*S*-hydroxy-5*Z*,8*Z*,10*E*,14*Z*-eicosatetraenoic acid-d_8
d_8-5(S)-HETE	5*S*-hydroxy-6*E*,8*Z*,11*Z*,14*Z*-eicosatetraenoic acid-d_8
d_4-(+/-)12,13-DiHOME	12,13-dihydroxy-9*Z*-octadecenoic acid-d_4

Table S2. Overview of the detected omega-3 and omega-6 oxylipins, including the variability observed in QC samples (expressed using RSD). Analytes showing an RSD < 30% in the QC samples were kept for further analysis.

	**Abbreviation**	**Name**	**RSD in QC samples**
Omega-3 oxylipins			
ALA-derived	ALA	α-linolenic acid	9.8%
	9-HOTre	9-hydroxy-octadecatrienoic acid	7.6%
	12,13-DiHODE	12,13-dihydroxy-octadecadienoic acid	5.8%
EPA-derived	EPA	Eicosapentaenoic acid	8.5%
	5-HEPE	5-hydroxy-eicosapentaenoic acid	13.1%
	12-HEPE	12-hydroxy- eicosapentaenoic acid	12.1%
	14,15-DiHETE	14,15-dihydroxy-eicosatetraenoic acid	8.0%
	17,18-DiHETE	17,18-dihydroxy-eicosatetraenoic acid	9.1%
	DPA	Docosapentaenoic acid	14.0%
DHA-derived	DHA	Docosahexaenoic acid	9.8%
	4-HDoHE	4-hydroxy-docosahexaenoic acid	14.3%
	8-HDoHE	8-hydroxy-docosahexaenoic acid	19.7%
	11-HDoHE	11-hydroxy-docosahexaenoic acid	17.1%
	13-HDoHE	13-hydroxy-docosahexaenoic acid	12.3%
	14-HDoHE	14-hydroxy-docosahexaenoic acid	14.7%
	16-HDoHE	16-hydroxy-docosahexaenoic acid	15.4%
	17-HDoHE	17-hydroxy-docosahexaenoic acid	9.0%
	20-HDoHE	20-hydroxy-docosahexaenoic acid	23.6%
	19,20-EpDPE	19,20-epoxy-docosapentaenoic acid	13.5%
	19,20-DiHDPA	19,20-dihydroxy-docosapentaenoic acid	7.9%
Omega-6 oxylipins			
LA-derived	LA	Linoleic acid	10.2%
	9-HODE	9-hydroxy-octadecadienoic acid	7.6%
	13-HODE	13-hydroxy-octadecadienoic acid	7.1%
	9,10,13-TriHOME	9,10,13-trihydroxy-octadecenoic acid	6.9%
	9,12,13-TriHOME	9,12,13-trihydroxy-octadecenoic acid	15.7%
	9,10-EpOME	9,10-epoxy-octadecenoic acid	7.8%
	9,10-DiHOME	9,10-dihydroxy-octadecenoic acid	7.3%
	12,13-EpOME	12,13-epoxy-octadecenoic acid	9.6%
	12,13-DiHOME	12,13-dihydroxy-octadecenoic acid	6.7%
	10-NO_2-LA	10-nitro-linolenic acid	13.3%
DGLA-derived	DGLA	Dihomo-γ-linolenic acid	23.9%
	8-HETrE	8-hydroxy-eicosatrienoic acid	22.8%
	15-HETrE	15-hydroxy-eicosatrienoic acid	13.5%
AA-derived	AA	Arachidonic acid	13.4%
	PGE_2	Prostaglandin E2	36.0%
	TxB_2	Thromboxane B2	6.9%
	12-HHTrE	12-hydroxy-heptadecatrienoic acid	8.1%
	8,12-$iPF_{2\alpha}$	8,12-isoprostane F2alpha	7.2%
	5-HETE	5-hydroxy-eicosatetraenoic acid	10.4%
	11-HETE	11-hydroxy-eicosatetraenoic acid	10.7%
	12-HETE	12-hydroxy-eicosatetraenoic acid	11.4%
	15-HETE	15-hydroxy-eicosatetraenoic acid	9.3%

	20-HETE	20-hydroxy-eicosatetraenoic acid	9.4%
	5,6-DiHETrE	5,6-dihydroxy-eicosatrienoic acid	9.0%
	8,9-DiHETrE	8,9-dihydroxy-eicosatrienoic acid	9.9%
	11,12-DiHETrE	11,12-dihydroxy-eicosatrienoic acid	8.4%
	14,15-EpETrE	14,15-epoxy-eicosatrienoic acid	19.5%
	14,15-DiHETrE	14,15-dihydroxy-eicosatrienoic acid	7.1%
AdrA-derived	AdrA	Adrenic acid	22.1%
	1a,1b-dihomo-$PGF_{2\alpha}$	1a,1b-dihomo-9S,11R,15S-trihydroxy-5Z,13E-prostadienoic acid	22.1%

Abbreviations: QC: quality control; RSD: relative standard deviation.

Table S3. Partial Spearman correlation between total omega-3 and omega-6 oxylipins and the relative abundance of fecal microbiota composition at genus level.

		Model 1		Model 2	
**Omega-3 oxylipins**					
		Rho	P	Rho	P
Clostridium cluster IV (%)					
	Σ Omega-3	0.286	**0.009**	0.287	**0.010**
Sutterella (%)					
	Σ Omega-3	-0.254	**0.022**	-0.255	**0.023**
**Omega-6 oxylipins**					
		Rho	P	Rho	P
Clostridium cluster IV (%)					
	Σ Omega-6	0.114	0.295	0.118	0.296
Sutterella (%)					
	Σ Omega-6	-0.265	**0.017**	-0.265	**0.017**
**Omega-6/omega-3 ratio**					
		Rho	P	Rho	P
Clostridium cluster IV (%)		-0.256	**0.021**	-0.256	**0.022**
Butyricimonas		-0.238	**0.033**	-0.238	**0.033**

Rho and P values are provided from partial Spearman correlation analyses. Model 1: adjusted for BMI; Model 2: adjusted for BMI, PUFAs and fish intake. The sum (Σ) of omega-3 and omega-6-derived oxylipins were computed from the individual area peak ratio of each oxylipins group. *Abbreviations*: BMI: body mass index; PUFAs: polyunsaturated fatty acids.

Table S4. Partial Spearman correlation between plasma levels of individual omega-3 and omega-6 oxylipins and the relative abundance of fecal microbiota composition at genus level.

	**Model 1**		**Model 2**	
**Omega-3 oxylipins**				
	Rho	P	Rho	P
Clostridium cluster IV (%)				
DPA	0.332	**0.003**	0.271	**0.003**
DHA	0.321	**0.004**	0.262	**0.004**
8-HDoHE	0.339	**0.002**	0.300	**0.002**
13-HDoHE	0.349	**0.002**	0.328	**0.002**
19,20-DiHDPA	0.237	**0.035**	0.160	**0.035**
Sutterella (%)				
ALA	-0.245	**0.029**	-0.224	**0.029**
EPA	-0.260	**0.020**	-0.241	**0.020**
5-HEPE	-0.363	**0.001**	-0.348	**0.001**
DHA	-0.231	**0.039**	-0.196	**0.049**
4-HDoHE	-0.287	**0.010**	-0.267	**0.010**
19,20-DiHDPA	-0.228	**0.042**	-0.202	**0.042**
**Omega-6 oxylipins**				
	Rho	P	Rho	P
Clostridium cluster IV (%)				
9,10-EpOME	-0.193	0.087	-0.198	0.087
12,13-EpOME	-0.257	**0.021**	-0.250	**0.021**
TxB_2	0.213	0.058	0.159	0.058
12-HHTrE	0.216	0.054	0.168	0.054
Sutterella (%)				
LA	-0.292	**0.009**	-0.278	**0.009**
DGLA	-0.260	**0.020**	-0.241	**0.020**
AA	-0.253	**0.024**	-0.233	**0.024**
5-HETE	-0.366	**0.001**	-0.351	**0.001**
AdrA	-0.311	**0.005**	-0.294	**0.005**

Rho and P values are provided from partial Spearman correlation analyses. Model 1: adjusted for body mass index; Model 2: adjusted for body mass index, PUFAs and fish intake. *Abbreviations*: BMI: body mass index; PUFAs: polyunsaturated fatty acids.

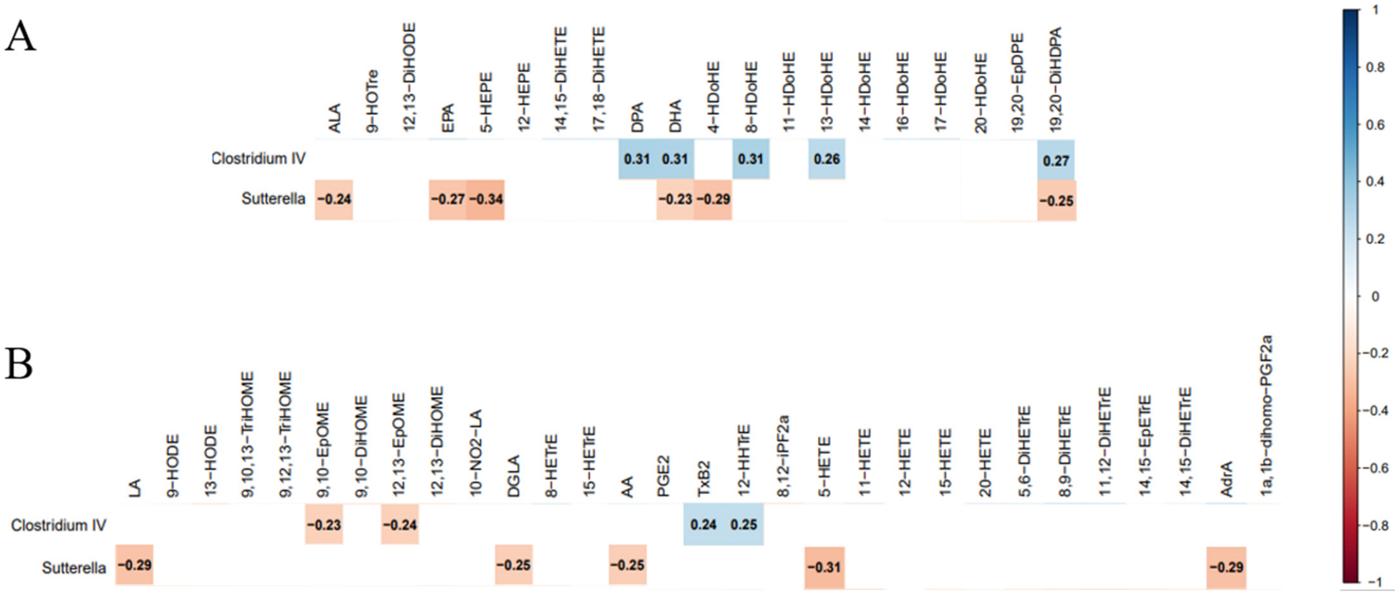

Figure S1: Spearman correlations between plasma levels of individual omega-3 (Panel A) and omega-6 (Panel B) oxylipins and the abundance relative of *Clostridium IV* and *Sutterella* genera. Boxes only represent the statistically significant ($P<0.05$) correlations and the value within the boxes show the Spearman correlation coefficient. Blue boxes represent positive correlations whereas red boxes indicate negative correlations.
